# Biosynthesis, Deficiency, and Supplementation of Coenzyme Q

**DOI:** 10.3390/antiox12071469

**Published:** 2023-07-21

**Authors:** Carmine Staiano, Laura García-Corzo, David Mantle, Nadia Turton, Lauren E. Millichap, Gloria Brea-Calvo, Iain Hargreaves

**Affiliations:** 1 Centro Andaluz de Biología del Desarrollo, Universidad Pablo de Olavide-CSIC-JA, 41013 Sevilla, Spain; csta2@upo.es (C.S.); lgarcor@upo.es (L.G.-C.); 2 Centro de Investigación Biomédica en Red en Enfermedades Raras (CIBERER), Instituto de Salud Carlos III, 28029 Madrid, Spain; 3 Departamento de Fisiología, Anatomía y Biología Celular, Universidad Pablo de Olavide, 41013 Sevilla, Spain; 4 Pharma Nord (UK) Ltd., Morpeth NE61 2DB, UK; dmantle@pharmanord.co.uk; 5 School of Pharmacy and Biomolecular Sciences, Liverpool John Moores University, Merseyside L3 5UX, UK; n.m.turton@2020.ljmu.ac.uk (N.T.); l.millichap@pm.univpm.it (L.E.M.)

**Keywords:** Coenzyme Q, CoQ biosynthesis, CoQ deficiency, blood-brain barrier, statins

## Abstract

Originally identified as a key component of the mitochondrial respiratory chain, Coenzyme Q (CoQ or CoQ_10_ for human tissues) has recently been revealed to be essential for many different redox processes, not only in the mitochondria, but elsewhere within other cellular membrane types. Cells rely on endogenous CoQ biosynthesis, and defects in this still-not-completely understood pathway result in primary CoQ deficiencies, a group of conditions biochemically characterised by decreased tissue CoQ levels, which in turn are linked to functional defects. Secondary CoQ deficiencies may result from a wide variety of cellular dysfunctions not directly linked to primary synthesis. In this article, we review the current knowledge on CoQ biosynthesis, the defects leading to diminished CoQ_10_ levels in human tissues and their associated clinical manifestations.

## 1. Introduction

Since its discovery in 1957 by Crane and collaborators as a redox-active mitochondrial lipogenic type compound [1], Coenzyme Q (CoQ), also known as ubiquinone (UQ), has increased remarkably in relevance. In subsequent years, it has become clear that this isoprenoid side chain-quinone is involved in the mitochondrial respiratory chain (MRC) of eukaryotes and many prokaryotes, being essential for oxidative phosphorylation. However, it was also soon recognised as an antioxidant, directly inhibiting lipid and lipoprotein peroxidation or indirectly protecting the plasma membrane by regenerating ascorbate [2] or alpha-tocopherol [3]. More recently, many other roles for CoQ have been discovered, as it is the electron acceptor for a number of different mitochondrial dehydrogenases. For instance, CoQ participates in various mitochondrial processes such as de novo pyrimidine biosynthesis, receiving electrons from dihydroorotate dehydrogenase, and the beta-oxidation of fatty acids and the oxidation of branched-chain amino acids by accepting electrons from the electron transport flavoprotein dehydrogenase (ETFDH) [4]. Thus, in the mitochondria, CoQ is the central element of the so-called Q-junction, where electrons coming from the MRC respiratory complexes I and II, the dihydroorotate dehydrogenase (DHODH), the choline and proline dehydrogenases (CHDH and PRODH respectively), the mitochondrial glycerol-3-phosphate dehydrogenase (G3PDH), the sulfide:quinone oxidoreductase (SQOR) and the previously mentioned ETFDH converge [4]. Electrons are then transferred from CoQ to MRC complex III in the inner mitochondrial membrane. In addition, CoQ has recently attracted increased interest from the scientific community, as it has been shown to participate in the FSP1 antioxidant system-mediated protection against ferroptosis, a form of regulated cell death caused by iron-dependent lipid peroxidation. Inhibition of the FSP1 antioxidant system is currently being studied as a promising approach to sensitise cancer cells to ferroptosis-inducing chemotherapeutic agents [5].

The redox properties of CoQ depend on its fully substituted benzoquinone ring head, which is linked to a highly hydrophobic polyisoprenoid chain that anchors the molecule to the midplane of the phospholipid bilayer [6]. The number of isoprene units varies among species, being mainly 10 in humans (CoQ_10_)-although also containing CoQ_9_ as a minor CoQ form-, 6 in *Saccharomyces cerevisiae* (CoQ_6_), and 8 in *Escherichia coli* (CoQ_8_). Mice have mainly CoQ_9_ but small amounts of CoQ_10_ as well. The different lengths of the hydrophobic chain are controlled by the particular size of the pocket in which the growing polyprenyl diphosphate is accommodated in the dedicated enzymes catalysing this step in the different species [7].

The two principal types of isoprenoid quinones found in all living organisms are CoQ and menaquinone (MK), which mainly differ in the structure of their respective head groups and the value of their redox midpoint potential. The CoQ head is a benzene ring (benzoquinone), while that for MK is a naphthoquinone [8]. MK is present in both *Archaea* and *Bacteria,* and CoQ is found in eukaryotes and only in some groups of proteobacteria. Interestingly, in addition to CoQ, rodent and human brains contain MK_4_ (vitamin K), which has been shown to act as an antioxidant defence and be involved in the inflammatory response [9]. A deficiency of MK_4_ has also been linked to cognitive dysfunction in rats [10]. MK is considered to be the quinone involved in the ancestral prokaryotic non-oxygenic respiratory chains. In contrast, it is proposed that CoQ would have evolved after the Great Oxidation Event, more than 2 billion years ago, being already present in the common ancestor of the alpha-, beta-, and gamma-proteobacteria as a more efficient quinone in an oxidative environment [11].

Thus, typically, MK and CoQ are thought to be involved predominantly in anaerobic and aerobic respiration, respectively [8,12]. However, the recent discovery of a widely conserved O_2_-independent CoQ biosynthetic pathway that could operate under anaerobic or microaerobic conditions suggests that CoQ may have a role also in anaerobiosis. Remarkably in proteobacteria, O_2_-independent CoQ biosynthesis coexists with the classical oxygen-requiring route. Both pathways differ only by three hydroxylation steps carried out by three different enzymes. Phylogenetic studies support the concept that these two systems are complementary and have evolved to allow the metabolic plasticity needed to face the wide range of environmental O_2_ levels experienced by facultative aerobes [12]. As the pathogenicity of certain bacteria relies on their anaerobic or microaerobic metabolisms, further exploration of the anaerobic CoQ biosynthesis would serve as a potential strategy to combat infectious diseases.

This review aims to outline our current knowledge of CoQ biosynthesis, together with the new advances made in this field. In addition, this review will provide information on primary and secondary CoQ_10_ deficiencies, therapeutic strategies to treat these disorders and blood–brain barrier (BBB) CoQ_10_ transport.

## 2. CoQ Biosynthesis 

CoQ can be obtained from the diet, but it is primarily endogenously synthesised within the mitochondrial inner membrane by a set of nuclear-encoded proteins (COQ proteins). Although CoQ is conserved from proteobacteria to humans, the diversity of CoQ biosynthesis pathways remains largely unexplored. Most information on eukaryotic CoQ biosynthesis derives from studies performed in bacteria and yeast. While in eukaryotes, CoQ biosynthesis likely starts in the cytosol and the endoplasmic reticulum, the main set of reactions are performed in the inner mitochondrial membrane by the COQ proteins, dynamically assembled in a complex (Q-complex or Q-synthome), that has not yet been wholly characterised [13]. Likewise, the mechanism by which CoQ is distributed to the rest of the membranes within the cell still needs to be completely deciphered. However, the lipid transfer protein STARD7, which—upon cleavage by the protease PARL—localises both in the mitochondrial intermembrane space and the cytosol, has been recently revealed as critical for CoQ distribution. While mitochondrial STARD7 ensures CoQ synthesis and thus oxidative phosphorylation, cytosolic STARD7 is essential for CoQ transport to the plasma membrane [14,15]. The mechanism by which CoQ distributes to other cell membranes is still unknown. In *E. coli*, a soluble cytosolic complex composed of several biosynthetic proteins has been demonstrated to perform some of the reactions, and then, translocate to the membrane to complete the process [16].

**Figure 1:**
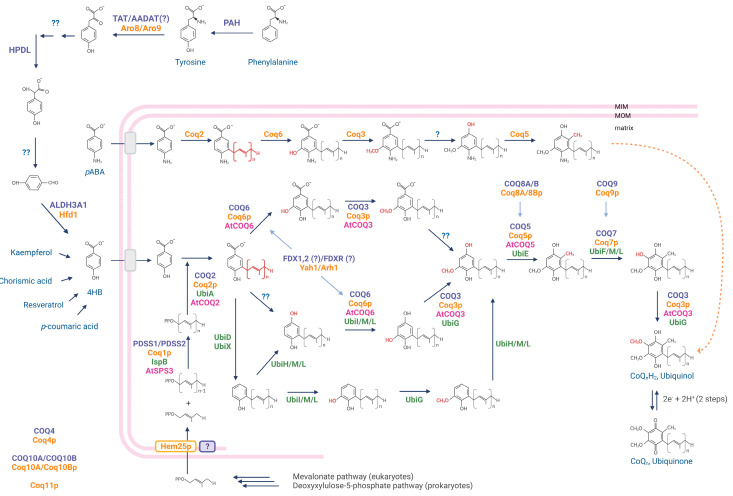
**CoQ biosynthesis pathway.** A compendium of the reactions carried out to synthesize CoQ in bacteria (enzymes in green), yeast (enzymes in orange), plants (enzymes in pink), and mammals (enzymes in purple) is depicted including the name of the proteins involved. Proteins whose functions are still unknown are indicated separately. For simplicity, only some proteins involved in the 4HB biosynthesis in mammals have been indicated. For an extensive revision on 4HB biosynthesis, see Fernández-del-Río and Clarke, 2021 [21]. PAH, phenylalanine hydrolase; TAT, tyrosine aminotransferase; AADAT, mitochondrial alpha-aminoadipate aminotransferase; ALDH3A1, aldehyde dehydrogenase 3A1; HPDL, hydroxyphenylpyruvate dioxygenase-like [22]; simbols ? and ?? indicate enzyme/s still not identified.

In general, the process of CoQ biosynthesis can be divided into four steps (Figure 1): (i) synthesis of the head precursor; (ii) synthesis of the side polyisoprenoid chain; (iii) condensation of head and tail; and (iv) sequential modification of the benzoquinone head ring. Both eukaryotes and prokaryotes use 4-hydroxybenzoate (4-HB) as the quinone head precursor. In prokaryotes, 4-HB is synthesised from chorismate [17]. In eukaryotes, it derives instead from tyrosine and possibly phenylalanine by a yet not wholly understood pathway that is probably carried out in the cytosol [18]. Most of the CoQ head precursor in plants comes from the beta-oxidation of *p*-coumarate in peroxisomes [19]. However, about a quarter of the 4-HB derives from the metabolism of the flavonol kaempferol, and there is also evidence supporting that some 4-HB also originates from tyrosine [20]. 

Of remarkable interest is the demonstration that mammalian cells can also use exogenous kaempferol as an aromatic ring precursor for CoQ biosynthesis, particularly in the kidney [23]. In addition to 4-HB, para-aminobenzoic acid (pABA) can also be a precursor of CoQ in yeasts. Conversely, in mammalian cell cultures, pABA behaves as a competitive inhibitor of the biosynthetic pathway [21]. In eukaryotes, archaea and some eubacteria, the isoprenoid units that build the CoQ hydrophobic chain are derived from the mevalonate pathway, which CoQ shares with cholesterol and dolichol biosynthesis. Very recently, Hem25p has been revealed as an isopentenyl pyrophosphate (IPP) transporter in *Saccharomyces cerevisiae,* although this function is only conserved in fungi [24]. Therefore, how isoprenoid units are transported into mitochondria still remains unclear. 

In any case, once in the mitochondria, the human heterodimer PDSS1/PDSS2, homologues of the polyprenyl diphosphate synthase Coq1p in yeast, catalyse the condensation of the isoprene units by a similar mechanism to that performed by the bacterial IspB to generate a polyprenyl diphosphate chain [25]. Importantly, the polyprenyl diphosphate synthase determines the size of the CoQ side chain [26]. After 4-HB is prenylated with the polyisoprenoid chain by the prenyltransferase Coq2p/COQ2 (UbiA in bacteria), sequential modifications of the head group are performed by other COQ proteins (Coq proteins in yeasts and Ubi proteins in bacteria). One decarboxylation in C1, the hydroxylation of C1, C5, and C6, and three methylations in C2, C5, and C6 are needed to obtain the final CoQ molecule. In *E. coli*, decarboxylation of C1 is catalysed by the UbiD-UbiX system. However, this step still needs to be fully characterised in eukaryotes, and it is unclear whether it occurs before, after or independently to the C5 hydroxylation [27]. Although three hydroxylations are needed in the biosynthesis of CoQ, in eukaryotes, only Coq6p/COQ6 and Coq7p/COQ7, modifying C5 and C6, respectively, are known. The hydroxylase for C1 has yet to be identified in this group. In bacteria, the hydroxylation of these three contiguous carbons in the head ring is performed by the flavin monooxygenases UbiH, UbiI, and UbiF/Coq7, or the recently discovered UbiM and UbiL, which have been shown to hydroxylate all three positions in some cases [28]. Almost 20 different combinations of these six hydroxylases have been identified in proteobacteria, highlighting the diversity of strategies to synthesise CoQ in this group [28]. Finally, while Coq3p/COQ3/UbiG O-methylates both C5 and C6, Coq5p/COQ5/UbiE is responsible for the C-methylation in C2 of the ring. Remarkably, UbiE is also involved in the biosynthesis of MK in bacteria, highlighting the crosstalk between the two biosynthetic pathways in prokaryotes [6,29,30,31,32,33].

Most of the research on CoQ biosynthesis in plants has been conducted in *Arabidopsis thaliana,* although some information comes from tomato. In *Arabidopsis thaliana*, AtSPS3 has been functionally determined to be the orthologue of Coq1 polyprenyl diphosphate synthase [34]. The *AtPPT1* gene, instead, codes for the protein transferring the polyprenyl side chain to the benzoquinone head [35]. Functional complementation in yeast has demonstrated that *AtCOQ3*, *AtCOQ4*, *AtCOQ5*, *AtCOQ6*, and *AtCOQ8 A. thaliana* genes are able to rescue the corresponding *S. pombe* mutants. Conversely, *AtCOQ9* is unable to complement *S. pombe* deleted strain. On the other hand, the protein performing the C6 hydroxylation in plants remains to be identified [31].

Other proteins with regulatory or accessory roles participate in CoQ biosynthesis. For example, although no enzymatic activity has been identified for COQ4, it is essential for CoQ biosynthesis, possibly allowing Q-synthome stabilisation [36]. Coq8p/COQ8A/COQ8B is a member of the UbiB kinase-like family of proteins that lacks kinase activity in vitro but shows ATPase activity. Coq8p has been proposed to extract the quinone heads of CoQ intermediates out of the membrane allowing their chemical modification [37]. Coq9/COQ9 is a lipid-binding auxiliary protein known to functionally interact with COQ7, both cooperating to access to the hydrophobic precursors and facilitating the set of modifications leading to the final CoQ molecule [13]. The human lipid-binding proteins COQ10A and B (in yeast Coq10p) are not essential for CoQ biosynthesis. Instead, they have been proposed to behave as chaperone-like proteins, guiding CoQ to the appropriate locations within the mitochondrial inner membrane [38]. Ferredoxin (Yah1) and ferredoxin reductase (Arh1) have been described to assist Coq6 in yeast [39]. However, it is still unclear whether the mammalian homologs have the same function. Coq11, whose exact role is currently unknown, was identified as essential for CoQ biosynthesis in yeast, but mammals have no orthologue for this gene [40,41]. Oct1 and Puf3, meanwhile, post-transcriptionally modify Coq5 in yeast [42,43,44]. In addition, it has been recently shown that CoQ biosynthesis—and not only distribution—depends on the PARL-mediated processing of STARD7 in mammals [15]. Whether there are other proteins primarily involved in CoQ biosynthesis is currently unknown and needs to be further investigated.

Detailed information about the organisation of the Q-synthome is currently lacking, including the components’ stoichiometry and regulation. However, a complex composed of the hydroxylase COQ7 and the lipid-binding protein COQ9 has been thoroughly characterised both structurally and functionally. According to this recent work, a double heterodimer formed by the two proteins would reshape the mitochondrial inner membrane allowing the accessibility of substrates to the proteins’ lipid-binding sites [13]. COQ9 would assist COQ7 to facilitate substrate delivery, although more research is required to clarify the precise molecular process by which COQ9 assists COQ7.

It is well known that cell organelles are not isolated from each other but, instead, are tightly packed together through membrane contact sites. Mitochondria and endoplasmic reticulum contact sites are maintained by tethering structures called ERMES (endoplasmic reticulum-mitochondria encounter structure complex). These regions mediate the exchange in different molecules between the two compartments. Functional and spatial coordination between the Q-synthome and the ERMES has been recently demonstrated. In fact, yeast and human head-modifying CoQ pathway proteins co-localise to multiple domains in the inner mitochondrial membrane in vivo, in close proximity to the mitochondria-ER contact sites [45]. Yeast ERMES null mutants undergo destabilisation of the CoQ_6_ biosynthetic complex, causing decreased CoQ_6_ levels and distribution, highlighting a functional association between them [46]. The spatio-functional association between ER-mitochondria contact sites and the CoQ domains would then facilitate substrate accessibility for efficient CoQ generation and distribution in cells. CoQ biosynthesis intermediates have also been proposed to be essential for domain formation [45]. The involvement of Coq11 in the ERMES-CoQ domains’ functional association is currently under investigation [40]. 

Incomplete data about extramitochondrial CoQ biosynthesis have also been reported. Although also involved in menaquinone-4 (vitamin K2) biosynthesis, UBIAD1 is a prenyltransferase shown to be required for CoQ biosynthesis in the Golgi apparatus. Data support that extramitochondrial CoQ biosynthesis would be essential for membrane redox signalling and protection from lipid peroxidation in the cardiovascular tissue [47]. The recent discovery of STARD7-mediated CoQ transport from the mitochondria to the plasma membrane raises the question of the importance of the role of extramitochondrial CoQ biosynthesis. As suggested by Heeringa et al., the Golgi-localized pool of CoQ could be relevant in specific cells and tissues as an essential antioxidant for plasma membrane lipids that are typically derived from the Golgi apparatus [48].

## 3. Deficiency of Coenzyme Q_10_

Impaired CoQ_10_ levels in tissues are the biochemical hallmark of the disorders collectively known as CoQ_10_ deficiency syndrome. These disorders can be caused either by mutations in the genes participating in the CoQ_10_ biosynthesis (*COQ* genes) or by defects not directly linked to this pathway, but rather related to other processes. The former disorders are classed as primary CoQ_10_ deficiencies [49]—and will be discussed in the following section—and the latter disorders are known as secondary CoQ_10_ deficiencies. Secondary deficiencies are caused by genetic or non-genetic defects, which can be related to oxidative phosphorylation (OXPHOS), other mitochondrial processes, or even non-mitochondrial cellular functions [50,51], and these will be discussed separately (see below). 

When considering CoQ_10_ deficiency, it is of interest to compare the corresponding situation with regard to vitamin D3, another essential nutrient endogenously synthesised, for which a whole program of detection has been implemented in the UK health system. In the UK, identification of vitamin D3 deficiency is readily established in the health system, with assays carried out routinely by hospital biochemistry departments. By contrast, the prevalence of CoQ_10_ deficiency in the general population is unknown, any routinary testing for CoQ_10_ deficiency is conducted, and CoQ_10_ assays are carried out by a relatively small number of specialised laboratories, usually based in universities or other research centres, hampering the detection of putative deficiencies. There is no recommended daily intake for CoQ_10_, and no programme to supplement CoQ_10_ in the general population. The identification of vitamin D3 deficiency via a standard blood test is well established; however, whether CoQ_10_ plasma levels reflect other tissues’ status has been questioned. This is partly because, contrarily to other tissues, which rely on the novo biosynthesis to maintain their CoQ_10_ status, plasma CoQ_10_ content depends on liver biosynthesis and dietary supply, being determinant of the plasma lipoprotein concentration. Lipoproteins are the primary carriers of CoQ_10_ in circulation. Approximately 58% of total plasma CoQ_10_ is estimated to be associated with the low-density lipoprotein (LDL) fraction. In this regard, to consider the level of circulatory LDL, the recommendation is to express plasma CoQ_10_ content as a ratio relative to either total plasma cholesterol or LDL-cholesterol [52].

## 4. Primary CoQ_10_ Deficiencies 

Primary CoQ_10_ deficits are autosomal recessive conditions characterised by a wide range of clinical symptoms [53], brought on by biallelic mutations in any of the *COQ* genes, which biochemically result in decreased levels of CoQ_10_ in tissues. The mainly affected organs in primary CoQ deficiencies are the central and peripheral nervous system, heart, skeletal muscle, and kidney [53]. However, the symptoms and severity of CoQ_10_ deficiency can vary widely depending on the specific mutation and the affected genes.

The diversity of symptoms and the lack of conclusive genotype–phenotype associations make the diagnosis of CoQ_10_ deficiencies challenging. However, for patients, it is essential to obtain an early diagnosis to enable appropriate treatment to start as soon as possible to delay or halt the progression of clinical manifestations. Conventionally, to detect the reduction of CoQ_10_ in tissues or fluids, biochemical tests are conducted when a CoQ_10_ deficiency is suspected. Nonetheless, CoQ_10_ plasma determination is a poor indicator for diagnosis since it can be influenced by the diet [54]. Instead, reliable biochemical determination of CoQ_10_ levels requires invasive skin or muscle biopsy. In the last few years, the widespread use of next-generation sequencing (NGS), either exome (WES) or whole-genome (WGS) sequencing [55] has allowed for the genetic diagnosis of CoQ_10_ deficiency. Therefore, a growing number of patients with this syndrome have been molecularly diagnosed, and new disease phenotypes caused by genes involved in the CoQ_10_ biosynthetic pathway have been revealed [56]. In recent years, thanks also to the NGS technology, patients harbouring pathogenic variations in *PDSS1*, *PDSS2*, *COQ2*, *COQ4*, *COQ5*, *COQ6*, *COQ7*, *COQ8A*, *COQ8B*, and *COQ9* have been identified and linked to a primary CoQ deficiency. Any patient harbouring defects in *COQ3* has not been identified to date, probably because of the severity of the associated defects. The highest number of patients with primary CoQ defects have been reported to harbour pathogenic variants in COQ8A and COQ8B [49]. 

Traditionally, primary CoQ_10_ deficiencies used to be classified into five clinical phenotypes: (i) encephalomyopathy (recurrent myoglobinuria, encephalopathy, and mitochondrial myopathy); (ii) cerebellar ataxia (cerebellar atrophy associated with other neurologic manifestations and, occasionally, endocrine dysfunctions); (iii) severe infantile multisystemic disease; (iv) nephropathy; and (v) isolated myopathy [57]. However, as next-generation sequencing has been more broadly implemented and new patients have been identified, it has become clear that the disease can actually manifest with a wide variety and combination of symptoms and different ages of onset, although most of these manifest during early infancy [49]. The infantile-onset multisystem phenotypes of CoQ_10_ deficiency have been recently redefined, and four phenotypic groups have been delineated: (i) isolated steroid-resistant nephrotic syndrome (SRNS), or SRNR accompanied by neurological involvement (found in patients with defects in *PDSS2*, *COQ2*, *COQ6*, or *COQ8B* (with later age-of-onset for *COQ8B*); (ii) encephalomyopathy, hypertrophic/dilated cardiomyopathy, lactic acidosis, and tubulopathy (associated with defects in *PDSS2*, *COQ2*, *COQ7*, or *COQ9*); (iii) neonatal cardio-encephalopathies (*COQ2*, *COQ4*, or *PDSS1*); and (iv) pure neurological syndromes, including isolated or combined Leigh syndrome, ARCA (autosomal recessive cerebellar ataxia), and refractory epilepsy (*COQ2*, *COQ4*, *COQ5*, *COQ7*, or *COQ9*) [55]. Currently, it is generally accepted that isolated myopathy is rather associated with secondary deficiencies and not with primary ones [49].

When there is a deficiency of CoQ_10_, not only the MRC, but many other cellular processes are potentially hampered, such as the antioxidant system or pyrimidine nucleotide synthesis, amongst others, which could explain the symptoms’ pleiotropy [58]. Although it is tempting to speculate that CoQ_10_ deficiency would secondarily impact ferroptosis due to the role of CoQ as a cofactor of FSP1 [5], there is still no clear data about such a connection. Remarkably, as for most mitochondrial diseases, there is still no definitive explanation for the great variety of manifestations among patients with pathogenic variants in different *COQ* genes. Symptoms associated with the central nervous system (CNS) have been recorded in patients with pathogenic variants in all of the disease-associated *COQ* genes, being less frequent in *COQ6* and *COQ8B* patients [49]. High levels of oxygen and energy are required for brain activity [59], and CoQ_10_ has a key role in the normal functioning of the nervous system [60]. Therefore, a variety of neurological symptoms may result from a CoQ_10_ deficiency. Manifestations such as seizures and encephalopathy, a condition that alters brain function or structure, are reported in *COQ2* [61], *COQ4* [62], *COQ5* [56], and *COQ9* [63] patients. On the other hand, the combination of seizures and ataxia, which result in inaccuracy, instability, and lack of coordination while performing voluntary movements, has been reported in COQ4 [64], *COQ5* [56], and *COQ8* [65] patients. Seizures are also described in *COQ9* probands [63,66]. *PDSS1* [61], *PDSS2* [67], and *COQ9* [68] mutations can result in encephalopathy as well. Progressive cerebellar atrophy and ataxia (ARCA2) is a neurodegenerative disorder characterised by an ataxic phenotype with movement disorders, sometimes accompanied by intellectual disability, seizures, tremor, dysarthria, dysmetria, dysdiadochokinesia, saccadic eye movements, dystonia, or spasticity. ARCA2 is found in most *COQ8A* patients [69]. All the members of the only family described to harbour a COQ5 pathogenic defect also manifest with a similar clinical phenotype [56]. Cerebellar ataxia has also been described in nearly half of the *PDSS2* patients [67]. Finally, patients with *PDSS1* [61] and *COQ7* [70] gene mutations have shown signs of developing peripheral neuropathy, a disorder in which the peripheral nerves become damaged. Missense variants of PDSS1 have been shown to cause optic atrophy and sensorineural deafness [71].

Most of the patients identified with mutations in the *COQ4* gene develop childhood-onset ataxia [72,73,74]. However, Cordts et al. screened a series of in-house exome and genome datasets and found a cohort of patients with biallelic variants in *COQ4* causing adult-onset neurologic symptoms [75]. The cause of the differential age-of-onset of *COQ4* patients remains unknown. Importantly, clinical and neuroimaging data analysis from patients with *COQ4* defects reveals that brain regions are specifically susceptible to mutations in this gene [76]. 

Although the main affected system is the CNS, functional studies are normally performed in muscle samples or skin fibroblasts, which have been demonstrated to manifest the molecular defects in patients. However, defects in *COQ* genes are not always accompanied by a biochemical CoQ_10_ deficiency, at least in certain tissues. Recently, new patients harbouring variants in the *COQ4* gene have been identified to display ataxia and motor impairment with normal CoQ_10_ levels in fibroblasts. This study demonstrated the pathogenicity of COQ4 dysfunction and its consequences on the central nervous system in a transgenic zebrafish model. The authors engineered a coq4 CRISPR/Cas9 in the Tg (Neurod1:GCaMP6f) zebrafish line, and in vivo studies were performed. The results showed that the zebrafish model displayed a remarkable reduction of cells in the cerebellum, the brain area that controls swimming behaviour, and reduced locomotor activity [74].

It has been shown that CoQ_10_ levels and its redox state are variable among human tissues. Specifically, the brain exhibits variations in CoQ_10_ content between different brain regions in humans and rats [77,78]. Moreover, many markers of cell-type-specific mitochondria and mtDNA copy number in health and disease have been reported, which could also influence the cellular CoQ_10_ status [79,80]. Likewise, it could be speculated that CoQ_10_ levels could vary among cell types in the brain. This mitochondrial diversity may reveal functional and molecular diversity in the brain as well as differential susceptibility to CoQ_10_ deficiency.

The heart is one of the most energy-demanding organs in the body, and therefore, it relies heavily on efficient mitochondrial function and energy production. CoQ_10_ deficiency can cause cardiac symptoms. Cardiomyopathy, a condition where the walls of the heart become enlarged and weak, is reported in *COQ2* [81], *COQ4* [64], *COQ7* [70], *COQ9* [63], and *COQ8B* [82] patients. On the other hand, heart failure is another commonly reported symptom in patients with *COQ4* [64] and *COQ8B* [83] gene mutations.

Because CoQ is also essential for energy production in muscle [84], a deficiency can lead to weakness and muscle fatigue. Although isolated myopathy is rarely described in primary deficiencies, muscle weakness is reported in various patients with different *COQ* genes mutations: *COQ2* [85], *COQ6* [86], *COQ7* [87], *COQ8A* [88,89], and *COQ8B* [90]. Muscle fatigue is reported in patients with *COQ8A* [91] and *COQ8B* [83] gene mutations, and exercise intolerance has been associated with *COQ8A* mutations [65].

Steroid-resistant nephrotic syndrome (SRNS) constitutes childhood’s second most frequent cause of chronic kidney disease. This condition is often observed in primary CoQ_10_ deficiency patients with extrarenal manifestations such as sensorineural hearing loss or neurologic deficit [92,93]. Pathogenic variants of *PDSS1*, *PDSS2*, *COQ2*, *COQ6*, and *COQ8B* genes have been related to SRNS, alone or in combination with other symptoms [53]. Early use of CoQ_10_ supplementation has been shown to minimise proteinuria and slow the disease progression in monogenic SRNS associated with primary CoQ_10_ deficiency [94]. The timing of treatment is crucial to prevent irreversible damage, considering that once severe kidney—or central nervous system (CNS) and other tissues—damage is established, it cannot be recovered. This explains the urgent need to reveal comprehensive genotype–phenotype correlations and rapid diagnosis strategies for patients.

## 5. Factors Causing Secondary CoQ_10_ Deficiency

Secondary CoQ_10_ deficiencies are more common than primary deficiencies and result from causes other than defects in its biosynthesis. An idea of the prevalence of CoQ_10_ secondary deficiency can be gained by considering the factors causing this type of deficiency. Depletion of CoQ_10_ levels can result from defective variants in genes unrelated to CoQ_10_ biosynthesis, the effects of ageing, exercise, prescription-type medications, availability of lipoprotein bloodstream carriers, and illness as outlined below.

**Pathogenic variants in genes unrelated to CoQ_10_ synthesis:** As noted above, CoQ_10_ deficiency can result from mutations in genes not directly associated with CoQ_10_ biosynthesis. Secondary CoQ_10_ deficiency has been reported in patients with mitochondrial DNA (mtDNA) depletions [95], mutations or deletions [96,97], and in patients with mutations in *APTX* [98], *ETFDH* [99], *BRAF* [100], *ACADVL*, and *NPC* genes [101]. The *APTX* gene codes for the DNA strand-break repair protein aprataxin; aprataxin deficiency impairs mitochondrial function, independent of its role in mitochondrial DNA repair. The *APTX* gene mutations result in ataxia oculomotor apraxia type 1, secondarily associated with CoQ_10_ deficiency [98]. The *BRAF* gene encodes the protein B-Raf, which has a role in the regulation of cell division and cell growth. Patients with muscular hypotonia resulting from *BRAF* genetic mutation and CoQ_10_ deficiency may show remarkable improvement following CoQ_10_ supplementation [100]. The *ACADVL* gene encodes the enzyme very long-chain acyl-CoA dehydrogenase, deficiency of which results in impaired long-chain fatty acid beta-oxidation, resulting in symptoms of hypoglycaemia and lethargy; supplementation with a combination of CoQ_10_, L-carnitine and riboflavin reportedly results in symptomatic improvement [102]. The *NPC* gene encodes the protein intracellular cholesterol transporter, deficiency of which results in Niemann-Pick disease; CoQ_10_ levels are reportedly reduced in patients with the latter disorder [103], although the molecular mechanism behind this CoQ_10_ dysregulation remains unknown. An analysis of the proteome and transcriptome of muscle and adipose tissues from patients and a mouse model with insulin resistance revealed a decrease in the mevalonate/CoQ biosynthesis pathway, accompanied by a decline in CoQ levels. Depletion of CoQ drove complex II-dependent oxidative stress and adipocyte insulin resistance [104]. A comparative transcriptomic and proteomic analysis of five knockout mouse strains defective in different mtDNA expression factors revealed secondary CoQ deficiency, followed by a general decrease in CoQ levels [105].

As for other mitochondrial systems, mitochondrial import defects could potentially interfere with the COQ proteins import, leading to secondary CoQ_10_ deficiency [106]. Recently, *SPART*-defective cells have shown an evident impairment of mitochondrial nuclear-encoded protein import and a significant decrease in COQ7 and COQ9 proteins, leading to a severe decrease in CoQ_10_. CoQ_10_ supplementation rescued cellular ATP production to the same levels reached by the re-expression of wild-type *SPART*, suggesting CoQ_10_ treatment as a promising therapeutic approach for patients carrying pathogenic variants in *SPART* [107] and confirming the secondary CoQ_10_ deficiency as one of the pathomechanisms of the disease.

Surprisingly, only a few studies have linked the secondary CoQ_10_ deficiency with defects in pathways related to the Q-junction. Mutations in *ETFDH*, coding for the protein electron-transferring flavoprotein dehydrogenase, result in multiple acyl-CoA dehydrogenase deficiency (MADD), also known as glutaric aciduria types IIA-C. This disorder is associated with mitochondrial dysfunction, oxidative stress, and inflammation. Patients may have depleted levels of CoQ_10_ [99], and supplementation with a combination of CoQ_10_ (60–240 mg/day), riboflavin (a precursor in the synthesis of FAD, 100–300 mg/day), and L-carnitine (50–100 mg/day) may result in significant symptomatic improvement [108,109]. The underlying mechanisms by which defects in all these genes cause CoQ deficiency is unclear, but at least in some cases, the decrease in CoQ content has been suggested to be secondary to a decrease in mitochondrial mass [110].

**Ageing:** The tissue levels of CoQ_10_ are impaired as a consequence of ageing. In humans, the highest levels of CoQ_10_ are found in the brain, heart, and lung tissues and occur at approximately 20 years of age, followed by a continual decline thereafter [111]. The level of CoQ_10_ in heart tissue at the age of 80 years is only 50% of that at 20 years of age [111]. Soderberg et al. [112] described the substantial decrease in CoQ_10_ levels in various brain areas between the ages of 30 and 90 years. CoQ_10_ levels in the skin epidermis are also reported to decrease as a result of ageing [113,114,115].

**Exercise:** CoQ_10_ levels may be depleted by physical exercise. Trained athletes typically have lower plasma CoQ_10_ levels than untrained individuals, with heavy training leading to a particular decrease in plasma CoQ_10_ levels caused by an increased tissue uptake of the isoprenoid [116]. CoQ_10_ biosynthesis proteins in the skeletal muscle biopsies of healthy men showed no regulation in response to normal-volume endurance training. In contrast, high-volume endurance training induced a decrease in COQ3, COQ5, COQ7, and COQ9, suggesting that CoQ regulation may differ according to the volume of the exercise [117].

**Medication:** A number of medications may cause depletion of CoQ_10_ levels; these include statins, anti-depressants, contraceptives, and hormone replacement therapy. Statins reduce cholesterol synthesis by inhibiting the enzyme HMG CoA reductase, the rate-controlling enzyme of the mevalonate pathway, which is shared with CoQ_10_ synthesis. Clinical studies have found that treatment with statins reduces CoQ_10_ levels in both blood and muscle tissue, which in turn may be associated with statin-related adverse effects [118]. The interaction between CoQ_10_ and statins has been discussed in more detail elsewhere in this article. A different class of cholesterol-lowering medication, the fibrates, may also reduce circulatory CoQ_10_ levels; gemfibrozil has been found to decrease the serum level of CoQ_10_ in hyperlipidaemic patients when corrected for either total cholesterol or LDL-cholesterol levels [119].

A clinical study by Moreno-Fernández et al. [120] found the level of CoQ_10_ in peripheral blood cells to be significantly diminished in psychiatric patients compared to normal controls and significantly decreased further in psychiatric patients treated with the tricyclic antidepressant amitriptyline. In a study using a tumour cell line (H460 cells), treatment with amitriptyline induced a dose-dependent decrease in CoQ_10_ levels. The decreased CoQ_10_ levels were associated with the down-regulation of the expression of the *COQ4* gene, as well as decreased COQ4 and COQ6 protein levels [121].

A clinical study by Palan et al. [122] reported significantly decreased serum CoQ_10_ levels in a group of 15 pre-menopausal women who had been taking oral contraceptives for at least 6 months, compared to a group of 40 similarly aged women not taking contraceptives. A further study of 30 pre-menopausal women by Palan et al. [123] found serum CoQ_10_ levels were significantly impaired irrespective of the method of contraception used, i.e., oral, vaginal ring, or transdermal patch. Palan et al. [124] also reported significantly diminished serum CoQ_10_ levels in a series of 15 post-menopausal women receiving hormone replacement therapy, compared to a cohort of 33 post-menopausal women who were not receiving this treatment. Transdermal patch contraceptives can result in a greater than 50% reduction in serum CoQ_10_ levels, i.e., in treated women, it was determined a value of 0.30+/−0.10 µmol/L compared to the corresponding value of 0.69+/−0.20 µmol/L in non-treated subjects [123].

Bisphosphonates, which are prescribed for the treatment of osteoporosis, are another class of medicines that decrease CoQ_10_ levels, particularly for those patients receiving intravenously administered drug [125]. The reduction in CoQ_10_ levels by bisphosphonates is a result of the inhibition of the enzyme farnesyl pyrophosphate synthase, which catalyses a key step in the biosynthesis of CoQ_10_ [126].

**Illness:** The levels of CoQ_10_ in blood and other tissues are typically decreased in a number of disorders, including cardiovascular disease, neurological disorders, diabetes, liver disorders, pulmonary disorders, and reproductive disorders (reviewed by Mantle et al. [127]). For example, in patients with heart failure, depletion of both circulatory and cardiac tissue levels of CoQ_10_ have been reported; in cardiac biopsy samples, CoQ_10_ levels in patients with NYHA Class III and IV heart failure were 0.28+/−0.04 μg/mg tissue dry weight, compared to the level in normal cardiac tissue of 0.42+/−0.04 μg/mg tissue dry weight [128]. In patients with PD, depleted levels of CoQ_10_ have been reported in plasma, platelets and brain tissue [78,129]. The depletion of tissue CoQ_10_ levels may, in turn, result from increased CoQ_10_ catabolism caused by high levels of free radical-induced oxidative stress associated with these disorders.

CoQ_10_ levels may also be impaired following infection. In patients with COVID-19, blood platelet CoQ_10_ levels were significantly decreased [130]. In patients with influenza, blood CoQ_10_ levels are reportedly diminished in both seasonal and pandemic (H1N1) forms [131,132].

## 6. Statins and CoQ_10_

Statins target the liver and competitively inhibit the mevalonate pathway enzyme, HMG-CoA reductase, decreasing hepatic cholesterol synthesis [133]. Since their introduction in 1987 to treat hypercholesterolemia, statins have demonstrated an excellent safety profile. However, although rare, statin-associated muscle symptoms (SAMS) have been associated with statin therapy, presenting as myalgia, myositis, muscle weakness and, in rare cases, rhabdomyolysis; however, in many patients, SAMS may be more subtle and may occur with or without any elevation in the serum level of the skeletal muscle enzyme, creatine kinase (CK) [133]. In addition, statin treatment has also been associated with cognitive impairment, with reports of memory loss, insomnia, depression, and cerebellar ataxia [134,135]. Although it is uncertain whether all statins are able to cross the blood–brain barrier (BBB), animal studies have indicated that the lipophilic statins lovastatin and simvastatin have this capability [136]. Long-term statin therapy has also been associated with an increased incidence of heart failure with preserved ejection fraction (HFpEF) [137]. At present, the cause of the SAMS, cognitive impairment and HFpEF associated with statin therapy have yet to be fully resolved. However, in view of the reports of impaired oxidative phosphorylation together with the commonality of the cholesterol and the CoQ_10_ biosynthetic pathway, a statin-induced CoQ_10_ deficiency has been suggested as a major contributory factor [133,134,138]. Although a number of studies have reported evidence of a deficit in plasma/serum CoQ_10_ status following statin treatment [133], few studies have directly assessed the effect of statin therapy on skeletal/cardiac muscle or cerebral (using a surrogate such as cerebrospinal fluid; CSF) CoQ_10_ status. In one such study, Päivä et al. reported a 34% decrease of skeletal muscle CoQ_10_ status following treatment with a high dose of simvastatin (80 mg/day) for 8 weeks [139].

**Figure 2:**
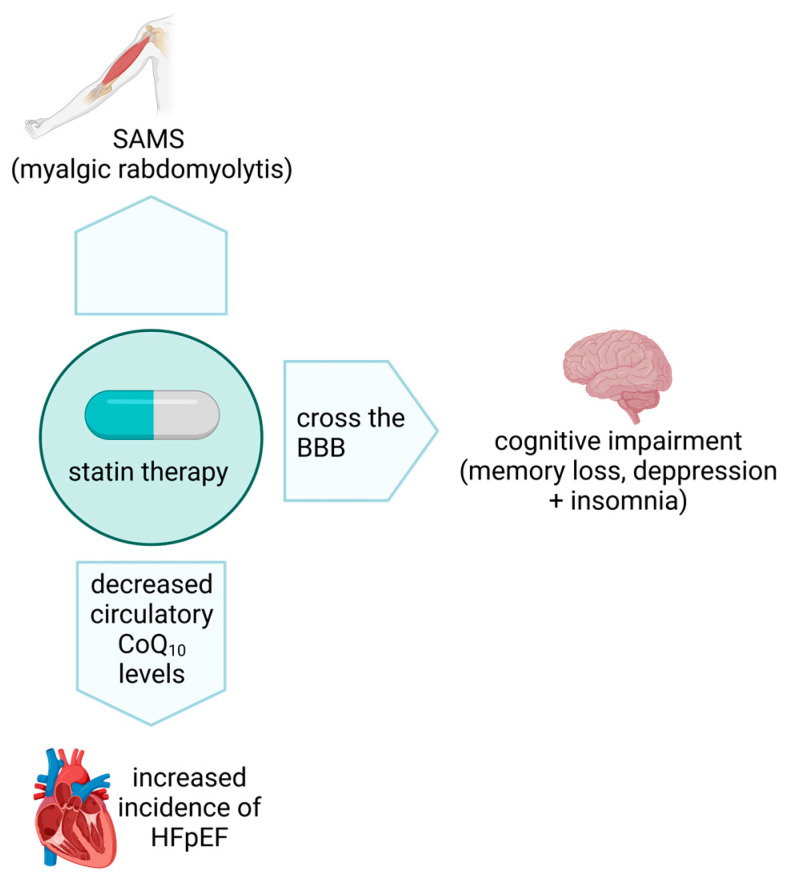
**Statin associated side effects.** SAMS—Statin associated muscle symptoms; HFpEF—Heart failure with preserved ejection fraction; BBB—Blood–brain barrier.

However, the development of cerebral ataxia following statin therapy [135,140] which is the most common clinical presentation of primary CoQ_10_ deficiency [140], has provided some possible support for the association between statin-induced neurological dysfunction and cerebral CoQ_10_ deficiency. Unfortunately, no assessment of CoQ_10_ status was made in the studies which reported an association between statin therapy and cerebral ataxia [135,140]. Figure 2 provides an overview of statin associated side effects.

Analysis of the effect of CoQ_10_ supplementing heart failure (HF) patients in the European cohort (231 of 420 patients) of the Q-SYMBIO study showed that the relative risk of MACE (major adverse cardiovascular events) was reduced by 67%, cardiac-related mortality by 53%, and all-cause mortality by 55%; in addition, left ventricular ejection fraction was significantly improved by 6% [141], thus indicating that increased plasma levels of CoQ_10_ provided clinical benefit for HF patients. However, since the major carriers of CoQ_10_ in the circulation are the LDLs, the continued lowering of these CoQ_10_ carriers in patients receiving long-term statin therapy may therefore deprive the myocardium of a supply of CoQ_10_ and contribute to the development of HFpEF in a cohort of patients receiving this pharmacotherapy [142].

## 7. CoQ_10_ and Blood-Brain Barrier Transport

CoQ_10_ primary deficiencies are one of the few mitochondrial conditions potentially treatable with high-dose CoQ_10_ supplementation during the early stages of the disease to limit irreversible damage in tissues. However, peripheral abnormalities may be improved by supplementation, but neurological symptoms are only partially or temporarily alleviated [143]. The reasons behind this limited response to CoQ_10_ supplementation are currently unknown. Nevertheless, its high molecular weight, hydrophobicity, and poor transfer across the BBB may be crucial contributory factors. Analyses evaluating how CoQ_10_ moves across the human BBB or whether a BBB CoQ_10_ deficiency may prevent CoQ_10_ from moving into the CNS are still needed. Nonetheless, a certain degree of CoQ_10_ transport across the BBB has been verified in some animal models. A 30% increase of CoQ_9_ (the main CoQ in rats) and CoQ_10_ in the cerebral cortex of 12-month-old Sprague–Dawley rats supplemented with CoQ_10_ (200 mg/kg) for 2 months has been reported [144]. In addition, high-dose CoQ_10_ supplementation (1000–5000 mg/kg) in a mouse model of Huntington disease resulted in a significant increase in CoQ_9_ and CoQ_10_ levels in the brain [145]. Whether the cerebral uptake of CoQ_10_ would be enough to rescue the cellular and, specifically, mitochondrial levels of this molecule in cases of CoQ_10_ deficiency needs further investigation. In contrast, there are studies reinforcing the observation that only a limited amount of CoQ reaches the brain. The study by Bentinger et al. (2003), in which [^3^H]CoQ_10_ was administered to rats intraperitoneally, found that there was virtually no uptake of the radiolabelled quinone into the brain. Conversely, high concentrations of [^3^H]CoQ_10_ were found in the spleen, liver, and white blood cells of the animals [146].

**Figure 3:**
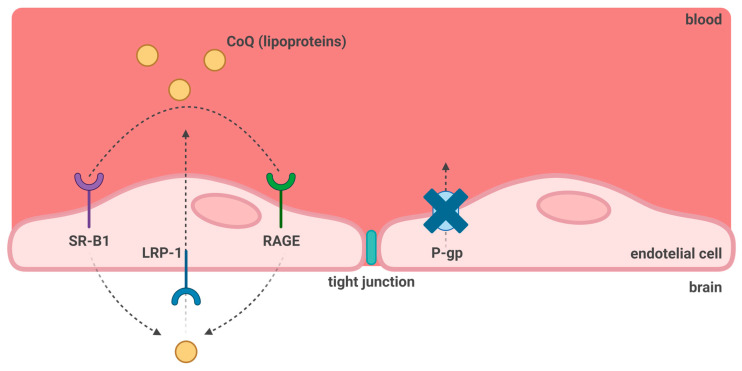
**CoQ_10_ transport across the blood–brain barrier under normal conditions.** SR-B1: Scavenger receptor B1; LRP-1: Low Density Lipoprotein related protein-1; RAGE: Receptor Advanced Glycation End-products; P-gp: P-glycoprotein transporter. Although P-gp has been shown to mediate CoQ_10_ transport in other cell lines, it does not participate in its movement across the BBB [147].

To investigate the mechanism by which CoQ_10_ may cross the BBB, Wainwright and colleagues (2020) [147], utilising a porcine brain endothelial cell model of the barrier, identified lipoprotein-associated CoQ_10_ transcytosis in both directions across the in vitro BBB. Uptake of CoQ_10_ via SR-B1 (Scavenger Receptor) and RAGE (receptor for advanced glycation end products) was found to be matched by CoQ_10_ efflux via LDLR (low-density lipoprotein receptor) transporters, resulting in no “net” transport of CoQ_10_ across the BBB [147] (Figure 3). When a CoQ_10_ deficiency was induced in the porcine model of BBB by treatment with para-aminobenzoate [148], the BBB tight junctions were disrupted, and the net transport of CoQ_10_ to the brain side of the barrier increased. Importantly, the study by Wainwright et al. implies that the uptake of exogenous CoQ_10_ into the brain may be improved by the administration of LDLR inhibitors or by interventions to stimulate the luminal activity of SR-B1 transporter. Unfortunately, the ability of CoQ_10_ supplementation to restore the damaged BBB following the pharmacologically induced CoQ_10_ deficiency was not assessed in the study by Wainwright et al., and this requires further investigation [147]. 

Interestingly, in a phase II clinical trial undertaken by Shults et al. [149], treatment of PD patients with daily doses of CoQ_10_ (300, 600 or 1200 mg) was found to result in a significant slowing of functional decline, indicating the possibility that CoQ_10_ was able to access the BBB, although the apparent therapeutic efficacy of CoQ_10_ supplementation may have been the result of beneficial effects of this molecule on the blood side of the BBB. However, in a subsequent phase III clinical trial in which PD patients were supplemented daily with CoQ_10_ (1200 mg + 2400 mg) together with vitamin E (1200 IU), no evidence of any clinical benefit to patients was reported [150]. The possibility arises that co-administration of a high dosage of vitamin E could have inhibited the transport of CoQ_10_ across the BBB via competition for the shared lipoprotein or other carrier types. Together with CoQ_10_, further studies are required to elucidate the mechanism(s) by which vitamin E is transported across the BBB [151].

In order to facilitate the transport of CoQ_10_ across the BBB, both synthetic analogues such as idebenone and MitoQ, together with water-soluble formulations of CoQ_10_ such as Ubisol-Q_10_ have been developed, although the safety and efficacy of these molecules have yet to fully evaluated in clinical trials [143,152]. 

## 8. Bypass Treatments for CoQ_10_ Deficiencies

Due to CoQ poor bioavailability, especially for the CNS, alternative strategies to reactivate CoQ endogenous biosynthesis are under investigation. Some 4-HB analogues with greater bioavailability than CoQ have been suggested as possible bypass molecules [153,154], providing the chemical group lacking due to defects in specific enzymes. 

2,4-dihydroxybenzoic acid (DHB), also called β-resorcilic acid (β-RA), was first demonstrated effective in a Δcoq7 yeast strain overexpressing Coq8 [155]. This C6-hydroxylated 4-HB molecule was later shown to circumvent *COQ7* and *COQ2* defects in patient-derived cell lines [70,87,156] and a *Coq7* knockout mouse model, in which a partial restoration of CoQ biosynthesis, mitochondrial respiration, abnormal phenotypic traits, and lifespan was demonstrated [157]. DHB has also been tested in two different *Coq9*-defective mouse models showing divergent effectiveness probably determined by the levels of COQ biosynthetic proteins and the integrity of the Q-synthome [158]. A follow-up study analysing the DHB effect on the *Coq9^R293X^* mouse model demonstrated the rescue of the biochemical, histopathological, and clinical phenotypes and suggested that the bypass treatment effectiveness depends on decreasing the levels of the potentially toxic intermediary DMQ rather than on the increase in CoQ levels. Therefore, according to the authors, DHB should be preferentially considered as a treatment for DMQ-accumulating defects [159]. A podocyte-specific *Coq6* knockout mouse (*Coq6*podKO) has been recently shown to alleviate proteinuria, avoid focal segmental glomerulosclerosis, and increase survival upon DHB treatment [160]. DHB was also demonstrated to prevent FSGS in a podocyte-specific *Adck4* (*Coq8B*) knockout mouse model, which recapitulates the clinical features described in *ADCK4* patients. Decreased CoQ levels and respiration defects in *Adck4*-defective podocytes derived from this mouse model were rescued by DHB treatment, demonstrating that the phenotypes were caused by defects in CoQ biosynthesis and contributing to the advance to alternative bypass treatments to treat nephrotic syndrome caused by *Adck4* dysfunction [161].

3,4-dihydroxybenzoic acid, a C5-hydroxylated form of 4-HB, rescues CoQ_6_ biosynthesis, and respiration in a *Coq6*-defective yeast strain [162]. On the other hand, vanillic acid (VA) is a C5-methoxylated form of 4-HB which has been demonstrated to bypass *Coq6* defects in yeasts and COQ6-defective cell lines [27,162,163]. VA, however, only partially rescued the migration defect observed in podocytes upon COQ6 knockdown by siRNA [160]. Recently, preclinical studies in another model of CoQ deficiency have demonstrated a positive effect of VA. VA treatment partially rescued the DMQ/CoQ ratio dysregulation observed in the *Coq9^R239X^* mouse model, mainly in the kidney. The level of mitochondrial bioenergetics improvement was determined by the tissue-specific response to VA, which was particularly efficient in the kidney but not in the brain. The same study also demonstrated that both DHB and VA treatments could correct the Q-junction disruption in the *Coq9^R239X^* mice, partially normalising the mitochondrial proteome and metabolome landscapes. These 4-HB analogues reversed the encephalopathic phenotype by reducing reactive gliosis, neuroinflammation, and spongiosis caused by the defect of CoQ metabolism in the *Coq9^R239X^* mouse model [164].

Although they have only been tested in yeast, mammalian cell cultures, and mouse models of primary CoQ deficiency, analogues of 4-HB are promising for patients’ treatment. However, further analyses are needed to understand their molecular mechanism of action and estimate their therapeutic response and safety. Nonetheless, several hydroxybenzoic acid compounds, especially DHB, are FDA- (EAF 3045, CAS RN 89–86–1) and EFSA- (Ref. no. 00910) approved food additives [160], making the bypass strategy promising for future clinical therapeutic applications.

## 9. Concluding Remarks

The elucidation of the CoQ biosynthetic pathway is providing some very interesting information about the Q-synthome, the importance of chaperones and regulatory proteins in the biosynthetic process together with the key role of the ERMES in the synthesis and cellular distribution of CoQ. An important knowledge gap has been recently closed, as STARD7 has been demonstrated to be essential for CoQ transport to the plasma membrane. However, at present, there is a paucity of information on the cellular uptake of exogenous CoQ_10_ in humans, whether it be via passive transport through the plasma membrane or via a form of endosome-mediated endocytosis. Importantly, this may have a bearing on the efficacy of supplementation-based therapeutic strategies used to treat disorders of CoQ_10_ deficiency. This is especially pertinent in the treatment of neurological disorders where the results from CoQ_10_ supplementation have generally been disappointing. Moreover, the ability of CoQ_10_ to cross the BBB or, indeed, access the cells of the brain in humans is still uncertain, and this may also have a bearing on future mediated gene therapies to treat primary CoQ_10_ deficiencies. However, the potential co-administration of LDLR inhibitors with CoQ_10_ supplementation may be an avenue to pursue in a bid to improve cerebral CoQ_10_ uptake. There are still a number of hurdles to overcome with the diagnosis and treatment of disorders of CoQ_10_ biosynthesis, confirming/refuting a genotype–phenotype relationship, deciding on the appropriate tissue required to establish a biochemical diagnosis and the ability to diagnose a primary CoQ_10_ deficiency within the early neonatal period before the disorder becomes irreversible and refractory to treatment, although the use of blood spot cards to determine CoQ_10_ status is under consideration.

## Data Availability

Not applicable.
